# Microbiological and Imaging-Based Evaluations of Photodynamic Therapy Combined with Er:YAG Laser Therapy in the In Vitro Decontamination of Titanium and Zirconia Surfaces

**DOI:** 10.3390/microorganisms12071345

**Published:** 2024-06-30

**Authors:** Ioana-Roxana Munteanu, Ruxandra-Elena Luca, Elena Hogea, Ralph-Alexandru Erdelyi, Virgil-Florin Duma, Liviu Marsavina, Amelia-Larisa Globasu, George-Dumitru Constantin, Darinca Carmen Todea

**Affiliations:** 1 University Clinic of Oral Rehabilitation and Dental Emergencies, Faculty of Dental Medicine, “Victor Babes” University of Medicine and Pharmacy, 300041 Timisoara, Romania; munteanu.roxana@umft.ro (I.-R.M.); todea.darinca@umft.ro (D.C.T.); 2 Interdisciplinary Research Center for Dental Medical Research, Lasers and Innovative Technologies, 300070 Timisoara, Romania; 3 Department XIV, Discipline of Microbiology-Virology, Faculty of General Medicine, “Victor Babes” University of Medicine and Pharmacy, 300041 Timisoara, Romania; hogea.elena@umft.ro; 4 Department of Measurements and Optical Electronics, Faculty of Electronics, Telecommunications and Information Technology, Polytechnic University Timisoara, 300006 Timisoara, Romania; ralph.erdelyi93@gmail.com; 5 Center of Research and Development for Mechatronics, National University of Science and Technology Politehnica Bucharest, 060042 Bucharest, Romania; 6 3OM Optomechatronics Group, Faculty of Engineering, “Aurel Vlaicu” University of Arad, 310177 Arad, Romania; 7 Department of Mechanics and Strength of Materials, Faculty of Mechanical Engineering, Polytechnic University Timisoara, 300222 Timisoara, Romania; liviu.marsavina@upt.ro; 8 University Clinic of Pedodontics, Faculty of Dental Medicine, “Victor Babes” University of Medicine and Pharmacy, 300041 Timisoara, Romania; a.amelialarisa@yahoo.com; 9 Department of Internal Medicine, Discipline of Clinical Skills, Faculty of General Medicine, “Victor Babes” University of Medicine and Pharmacy, 300041 Timisoara, Romania; george.constantin@umft.ro; 10 Advanced Cardiology and Hemostaseology Research Center, 300070 Timisoara, Romania

**Keywords:** dental implants, laser therapy, biofilm, photodynamic therapy, Er:YAG laser, titanium, zirconia, optical coherence tomography, scanning electron microscopy, *Staphylococcus aureus*

## Abstract

The oral cavity’s soft and hard tissues create a conducive environment for microbial proliferation and biofilm development, facilitating the colonization of prosthodontic and implant materials such as titanium (Ti) and zirconia (Zr). This study aimed to compare the efficacy of conventional decontamination methodologies (i.e., chemical and mechanical, using 0.12% digluconate chlorhexidine (CHX) solution-treatment and airflow) to adjunctive laser-based interventions on Ti and Zr substrates inoculated with *Staphylococcus (S.) aureus* ATCC 25923. Additionally, this investigation sought to elucidate the impact of these treatments on temperature variations and surface integrity, analyzing the laser irradiation effects on these prevalent dental materials. Experimental configurations were delineated for both Ti and Zr samples across four groups: (1) a conventional treatment group (CV); (2) a photodynamic therapy group (PDT); (3) an Er:YAG laser treatment group (Er); (4) a combined PDT and Er:YAG treatment group (PDTEr). Also, a negative control group (C) that received no treatment was considered. The decontamination of the inoculated disc samples was evaluated by quantifying the microbial colonies in colony-forming units per milliliter (CFU/mL). Temperature variations on the surface of the samples were determined during laser treatments. Surface modifications were investigated using scanning electron microscopy (SEM) and optical coherence tomography (OCT). For statistical analysis, Fisher 95% confidence intervals, Hsu’s MCB method, and the Kruskal–Wallis test were applied. With regard to the 10^5^ CFU/mL of the negative control group, results indicated average values equal for each study group to (1) 2.66 CFU/mL for Ti and 2 CFU/mL for Zr for the CV group; (2) 0.33 CFU/mL for Ti and 1 CFU/mL for Zr for the PDT group; (3) 1.25 CFU/mL for Ti and 0 CFU/mL for Zr for the Er group; (4), and 0 CFU/mL for both Ti and Zr for the PDTEr group. Therefore, the combined PDT and Er:YAG treatment (PDTEr) and the singular PDT modality outperformed conventional decontamination methods in eradicating *S. aureus* biofilms from both Ti and Zr surfaces. Notably, the PDTEr regime achieved a comprehensive elimination of microbial colonies on treated substrates. Surface examination employing OCT demonstrated discernible alterations in the surface morphology of samples subjected to Er:YAG and combined PDT and Er:YAG treatments. Temperature checks during treatments showed no major changes, suggesting the applied laser methods are safe. In conclusion, PDTEr and PDT eliminated bacteria more effectively, but Zr surfaces were more resilient, making them better for microbe-controlling applications. Also, the study demonstrated that the (less costly but lower resolution) OCT method can replace SEM for such investigations.

## 1. Introduction

Throughout the oral cavity, soft and hard tissues provide an ideal environment for microbial growth and biofilm formation that can spread to the surfaces of artificial materials utilized in restorative applications [1]. Because long-term dental materials are employed, it has become increasingly important to prevent infections and to reduce the risk of oral diseases such as dental caries, periodontitis, peri-implantitis, and mucositis. Dental structures and implants can alter the physiological, chemical, and mechanical environment of the oral cavity, thereby creating new niches for microbes [2]. Their susceptibility to contamination is mainly influenced by their topography and the surface chemistry of their material.

The popularity of dental implants has increased over the years. Today, they are the preferred treatment option for tooth replacement. One of their main issues is that intraoral supra- and subgingival structures are surrounded by gingival crevicular fluid, food debris, saliva, sugars, and bacterial metabolites. This creates a microenvironment that favors microbial growth, especially for Gram-negative pathogens that can cause peri-implantitis (PI) and periodontitis [3].

The majority of studies in the literature have shown that the composition of the subgingival microbiota associated with health and disease is similar around implants and teeth. Also, there has been emerging evidence that differences may be present in some of the peri-implant infections [4]. In vitro studies have demonstrated an affinity of *Staphylococcus (S.) aureus* for titanium (Ti) surfaces. Nevertheless, existing reports reveal the fact that the presence of *S. aureus* could be associated with therapy-resistant (i.e., refractory) cases of periodontitis as well [5].

Existing research has outlined the implications of *S. aureus* as being one of the most common bacterial pathogens involved in the early failure of dental implants because of its ability to attach to different Ti surfaces [6]. Important evidence states that bacterial species commonly associated with nosocomial infections and multi-resistance to antimicrobials (such as *Pseudomonas aeruginosa*, *S. aureus*, *Acinetobacter baumannii*, *Enterococcus faecalis*, and *the Enterobacteriaceae family*) have been detected in high proportions and levels in the subgingival biofilm of individuals with periodontitis [7]. Several studies have evaluated the implication of *S. aureus*, which is one of the most common causes of nosocomial and community-acquired infections in chronic and aggressive PI and periodontitis [8].

PI is a complication that already affects approximately 10% of implants and 20% of patients within 5 to 10 years of implant placement, according to a recent study [9]. In the development of PI, the formation of a biofilm on the surface of the implant plays a major etiological role. Also, PI diseases have been associated with the existence of bacterial plaque. Once these biofilms form on the implant surface, the removal of the bacteria and their byproducts, such as lipopolysaccharides, has been proven to be an issue. PI can lead to implant loss [10]. In the 1994 EWOP definitions, it was stated that mucositis was a reversible inflammatory reaction, while the term “reversible” was not included for PI [11]. The Consensus report of workgroup 4 of the 2017 World Workshop did not change much compared to the previous one. Thus, peri-implant mucositis, which is assumed to precede PI, is characterized by inflammation in the peri-implant mucosa and subsequent progressive loss of supporting bone. In the absence of treatment, it seems to progress in a non-linear and accelerating pattern. These definitions may imply that the inflammatory process occurring in PI lesions is irreversible. Therefore, it is not possible to treat them completely [12].

Given the closely aligned etiologies of periodontitis and PI infections, which are centered primarily on microbial involvement, therapeutic strategies require a comprehensive approach that targets this microbial component. Such an approach encompasses stringent oral hygiene practices and meticulous mechanical debridement of affected root surfaces in periodontitis management. The therapeutic regime for PI adheres to these principles as well, acknowledging poor oral hygiene as a critical risk factor for its development. For individuals presenting with concurrent untreated periodontitis and PI, the simultaneous treatment of both conditions is imperative, requiring the mechanical debridement of both contaminated implant and root surfaces in order to achieve optimal therapeutic outcomes [13]. While therapies aim to decrease the number of bacteria in the pockets around the implant and to clean the implant surfaces, in certain cases, they also attempt to stimulate bone regeneration.

A gold standard refers to a well-established and widely accepted material or method that can be used as a benchmark under normal circumstances, and that is known for its reliable reputation. While it may not be a flawless modality, it is the optimal option currently in use that adheres to established standards and produces predictable outcomes. Thus, for inflammatory periodontitis, periodontal debridement (PD) is considered the most effective treatment. This mechanical component of the therapy is correlated to the chemical one, using 0.12% digluconate chlorhexidine (CHX) solution, which has long been acknowledged as the primary agent for chemical plaque control [14]. It is regarded as the benchmark antimicrobial agent against which the effectiveness of other antimicrobial and antiplaque agents is evaluated. These two approaches, mechanical and chemical, define the conventional treatment that is utilized for the negative control group in the present study.

On the other hand, recent advancements have positioned laser application as a viable adjunct or alternative to the above conventional treatment modalities. Nevertheless, a thorough evaluation of potential adverse effects is essential prior to the clinical adoption of laser irradiation for implant surface treatment. Specifically, Er:YAG lasers have demonstrated efficacy in decontaminating Ti surfaces, albeit with mixed findings regarding their impact on the biological characteristics of such Ti surfaces and the potential thermal harm to adjacent tissues [15]. Concurrently, photodynamic therapy (PDT) has emerged as a beneficial bactericidal intervention in managing periodontal infections, underscoring its therapeutic potential [16].

To scrutinize the potential detrimental effects of laser irradiation on Ti or zirconia (Zr) surfaces, scanning electron microscopy (SEM) serves as a pivotal investigative tool [17]. While SEM remains the benchmark for surface analysis, recent studies have introduced optical coherence tomography (OCT) as a cost-effective alternative with good enough resolution [18,19,20,21,22], which is capable of replacing SEM in specific contexts, including the examination of metallic fractures [23,24] and the degradation of polymers [25].

The main aim of the present study was to evaluate the effectiveness of the laser therapy in the decontamination of both Ti and Zr surfaces by using PDT, Er:YAG laser, or a combination of both. For PDT, a laser diode (PACT^®^ 300, Cumdente GmbH, Tübingen, Germany) with a center wavelength of 635 nm was utilized. An Er:YAG laser (LightWalker ST-E, Fotona d.o.o., Ljubljana, Slovenia) with a center wavelength of 2094 nm was applied in the study.

Secondary aims were (i) to perform a comparison between the effectiveness of conventional mechanical and chemical treatments with the laser treatment applied to the surface contaminated with *S. aureus*; secondary aims were (ii) to perform a comparison between the effectiveness of conventional mechanical and chemical treatments, with or without adjuvant laser treatment applied to the surface contaminated with *S. aureus*; (iii) to assess eventual damages produced by laser radiation on the treated surfaces by using both SEM and OCT evaluations; (iv) to investigate if the less costly (although smaller-resolution) method. OCT can replace the common imaging method, SEM, for evaluating bacterial damage.

## 2. Materials and Methods

### 2.1. Study Design and Ethics

The research utilized Grade IV Ti alloy discs in accordance with ISO 22674, 98.5 mm in diameter and 8 mm in thickness (Colado CAD Ti5, Ti Al6 V4, Ivoclar Vivadent AG, Schaan/Liechtenstein) and Zr ZirCad Prime plates of 16 mm thickness 98.5 mm in diameter and 16 mm in thickness (Ivoclar Digital, Schaan, Liechtenstein), along with Columbia Agar plates supplemented with 5% sheep blood and *S. aureus* for microbial studies. *S. aureus* was purchased as a bacterial culture product in freeze-dried format (produced by Thermo Fisher Scientific Inc, Waltham, MA, USA, and imported by AMS 2000 Trading Impex SRL, Bucharest, Romania).

In this study, we categorized Ti and Zr discs into five distinct groups for analysis:

(1) A negative control group (C) comprised 4 discs inoculated with *S. aureus* and left untreated in order to establish a baseline microbial presence;
(2) A conventional study group (CV) involved 4 discs inoculated with *S. aureus* as well, which underwent conventional decontamination methods incorporating both mechanical and chemical techniques (using CHX-treatment and airflow);
(3) A PDT study group, for which 4 discs inoculated with *S. aureus* were subjected to decontamination using the PDT approach;
(4) An Er:YAG laser study group (Er) consisting of 4 discs inoculated with *S. aureus* and decontaminated using Er:YAG laser radiation with a central wavelength of 2094 nm;
(5) A combined therapy study group (PDTEr) including 4 discs inoculated with *S. aureus* and treated with a dual approach PDT and Er:YAG laser radiation.

Regarding the use of *S. aureus*, it is worth mentioning that studies have determined that the microbiota at Ti oral implants, which developed after surgical procedures, was similar to the microbiota found at nearby teeth. A multitude of bacterial species have been identified at peri-implant sites that were clinically healthy. Nevertheless, there were some variations in the occurrence of submucosal/subgingival bacterial species between the implant and neighboring tooth locations. The occurrence of various species, especially *S. aureus*, was more common at implant sites compared to bacterial species commonly linked to periodontitis [26], hence the choice of this specific bacteria for our study.

The research incorporated a comprehensive array of equipment for treatments and measurements, including sterile inoculating loops, swabs, test tubes, a photosensitizing agent (tolonium chloride solution), a Memmert IN110 incubator (Memmert GmbH + Co. KG, Schwabach, Germany), 0.12% CHX solution, and various dental prophylaxis tools. For analytical purposes, the study employed a Teledyne FLIR T640 Thermal Imaging Camera (Teledyne FLIR, Leeds, UK), a high vacuum FEI Quanta 250 scanning electron microscope (Thermo Scientific™ Quanta™, Hillsboro, OR, USA) with a secondary Everhard–Thornley electron detector, and an in-house developed Swept Source (SS) OCT system [22,23,24]. The latter two equipment ensured measurements and analyses of treatment effects on sample surfaces, performed in the 3OM Research Group (“Aurel Vlaicu” University of Arad and Polytechnic University of Timisoara).

### 2.2. Sample Preparation

**Figure 1:**
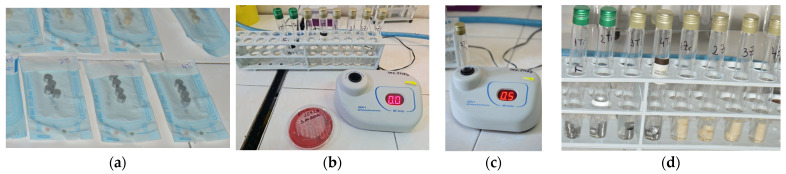
(**a**) Sterile Ti and Zr discs; (**b**) densitometer utilized for the suspension of *S. aureus* ATCC 25,923 0.5 Mc Farland; (**c**) harvesting *S. aureus* from Columbia Agar medium and checking the concentration of 0.5 CFU/mL using the McFahrad densitometer; (**d**) tubes with 4 mL of *S. aureus* suspension, having the samples immersed in a saline solution.

Thirty-two discs were milled using the CAD/CAM technique from a grade IV Ti alloy according to ISO 22674 (Colado CAD Ti5, Ti Al6 V4, Ivoclar Vivadent AG FL-9494 Schaan/Liechtenstein) and from Zr ZirCad Prime Ivoclar Digital. The standard dimensions for all discs were 8 mm in diameter and 4 mm in width—Figure 1a.

The processing of all samples was performed as presented in the phases shown in Figure 1.

Thus, the discs were sterilized in an autoclave at 134 °C for 30 min. Microbial cultures were performed: *S. aureus* was incubated on an agar blood culture medium (Columbia Agar + 5% ram blood) for 24 h at 37 °C in a thermostat. A suspension of *S. aureus* 0.5 Mc Farland was prepared. The 32 sterile Ti discs and Zr discs were immersed in 4 mL of *S. aureus* suspension in individual sterile containers, which were placed for 24 h at 37 °C in a thermostat. After incubation, the discs were subjected to a new sampling stage in order to assess the presence of *S. aureus* colonies. Confirmation test of bacterial fixation on biomaterials by transplanting on culture media was completed by scraping a portion of the culture on the surface of each substrate (Zr or Ti) with a calibrated loop of 1 μL and suspended with physiological saline. This suspension was readjusted at a concentration of 0.5 MacFarland. The samples were collected with a sterile loop and incubated on blood agar medium for 24 h at 37 °C in the same thermostat. Interpretative reading of cultures was made after incubation to quantify the microbial load.

For proper precision and control of the treatment protocols, as well as for reducing the possibility of bacterial contamination, each sample included in the study was fixed in a stand and isolated into an enclosure of plastic material, through which all treatments were applied. This ensured a reproducible position of the operator at the same distance from the sample, regardless of the applied treatment.

**Figure 2:**
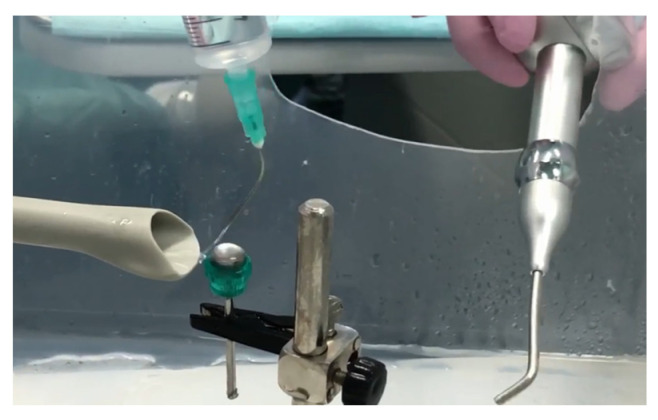
Surface irrigation with 2 mL of CHX 0.12% solution, followed by decontamination with airflow powder for 10 s using a PROPHY Mate M4 Prophylaxis Handpiece and KAVO PROPHYflex Prophylaxis Powder Perio Powder.

The next stage involved the treatment of each sample according to the specificity of each study group:

1. *Conventional study group (CV)*: Four discs inoculated with *S. aureus* were decontaminated using conventional methods, i.e., with two irrigation cycles for 10 s with 2 mL of CHX 0.12% solution, followed by airflow for 10 s using the hand for PROPHY Mate M4 prophylaxis and KAVO PROPHYflex Prophylaxis Powder Perio Powder (Figure 2).

The literature states that mechanical and chemical decontamination of the exposed implant surface with or without bone recontouring is essential, as it creates a healthy environment that allows for healing. The reason for choosing CHX solution for our study is based on previous studies [27], which recommended CHX for the treatment of contaminated Ti implant surfaces. It proved effective at killing bacterial cells and removing biofilm while causing little or no change to the surface characteristics of the Ti implant surface.

**Figure 3:**
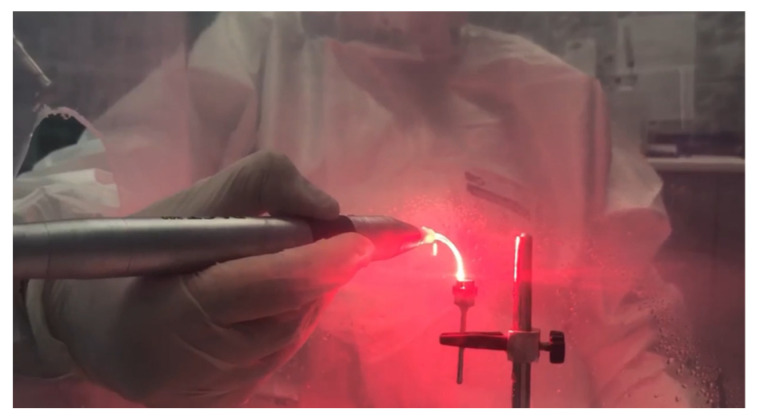
The 635 nm laser diode irradiation in 3 cycles of 10 s each by brushing movements at the level of the treated surface, with 10 s break between 2 successive irradiation cycles.

**Figure 4:**
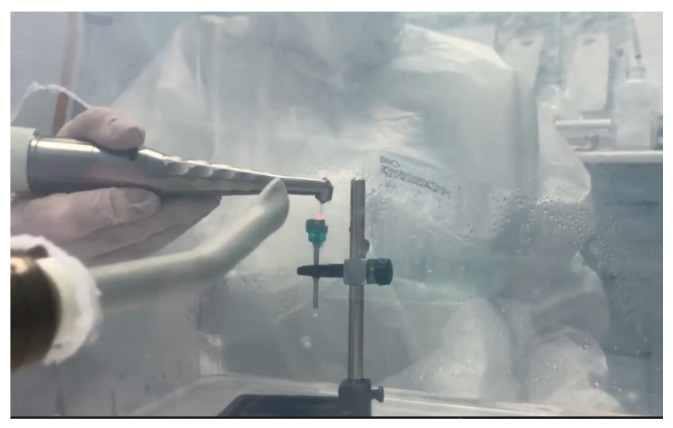
Irradiation with the Er:YAG laser through brushing movements at the level of the treated surface, oriented at an angle of 45°, and positioned at approximately 3 mm from the surface.

**Figure 5:**
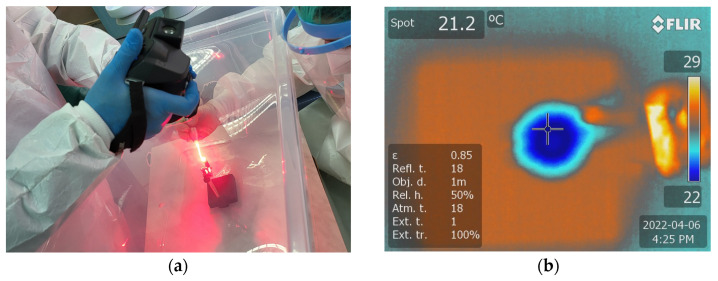
(**a**) Monitoring temperature variations during the laser treatment using a thermal camera FLIR T640 at (**b**) the level of the sample treated using both PDT and the Er:YAG 2094 nm laser.

2. *Study group PDT*: Four discs inoculated with *S. aureus* were decontaminated using the PDT method, as follows: immersion in photosensitizer dye liquid (Toluidine Blue Gel 0.005%, Cumdente) for 1 min, removal of excess solution with a light jet of air for 2 s, and finally, application of the diode laser. The treatment was performed using laser light at a wavelength optimized for photo-activation of the toluidine blue solution (635 nm), with a strong 400 mW (PACT 400-Cumdente) red light laser and a PACT Light Guide Universal (white) with spherical light emission for periodontal use, in 3 cycles of 10 s each [28] (although the irradiation time depends on the area of the treated surface), through brushing movements at the level of the treated surface, followed by a 10 s break, 90 s for each probe (Figure 3).

3. *Er:YAG study group (Er):* four discs inoculated with *S. aureus* were decontaminated using Er:YAG laser radiation centered at a wavelength of 2094 nm, with a 1.3 mm diameter and a 8 mm length quartz type, using the following settings: Super Short Pulse (SSP) or Quasi Super Pulse (QSP) mode, 100 mJ, 10 Hz, water 60, air 40, for 30 s, by brushing movements at the level of the treated surface, at an angle of 45°, at approximately 3 mm from the surface (Figure 4).

4. *PDT + Er:YAG (PDTEr) study group:* four discs inoculated with *S. aureus* were decontaminated using the combined method, using both PDT and Er:YAG laser radiation. The protocol steps implied an immersion in Toluidine Blue Gel 0.005%, Cumdente 60 s, rinsing with 2 mL saline, PDT irradiation in 3 cycles of 10 s each, with a 10 s break in between, followed by Er:YAG laser radiation using, as parameters, SSP mode, 100 mJ, 10 Hz, water 60, air 40, for 30 s (Figure 5a).

Temperature measurements were recorded from the center of the specimen during PDT and Er:YAG treatments, utilizing a FLIR T640 infrared thermographic camera. To prevent any measurement bias related to distance, the thermal camera was fixed at a distance of 25 cm from the discs. The discs underwent irradiation for a duration of 30 s, during which the temperature was continuously monitored. The thermal images were analyzed using SmartView software for Windows, version 1.6.0.5 to generate a thermal report and to determine the highest temperature of the disc (Figure 5b).

All decontamination treatments were performed on the same day for all samples by the same investigator and under the same environmental conditions in order to minimize human errors and to achieve a certain standardization of the working protocol.

**Figure 6:**
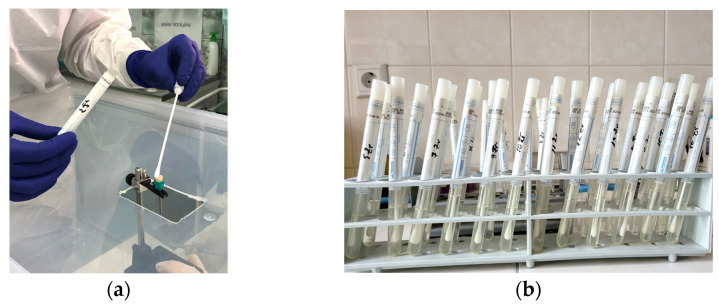
(**a**) Harvesting with a sterile swab from the treated surface of the samples; (**b**) storing of the culture media.

From the level of the treated surface of each disc, with the help of a sterile swab, samples were collected and stored in individual sterile tubes of pharyngeal exudate without medium. In each tube, 4 mL of sterile physiological serum was added. The tubes were stored after harvesting for 24 h at 37 °C, in a thermostat. For each disc included in the study, samples were collected using a sterile loop by brushing over the treated/decontaminated surface (Figure 6).

**Figure 7:**
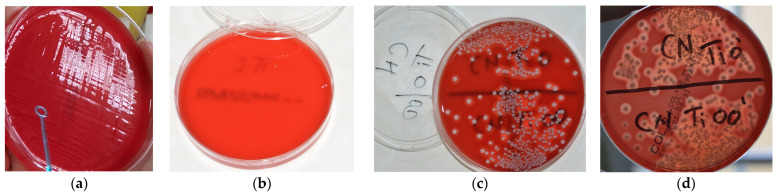
(**a**) Inoculation of the culture media for each individual sample; (**b**) the presence of two microbial colonies developed on the culture medium after 24 h of incubation at the thermostat for a sample collected from a Ti disc; (**c**,**d**) the number of colonies developed on the culture medium for the negative control samples quantified as 10^5^ CFU/mL.

After 24 h, each tube harvested from each treated sample was seeded on Columbia agar medium +5% ram blood. Further on, the culture media were stored for another 24 h at 37 °C in a thermostat. Subsequently, the colonies of *S. aureus* were counted. Quantification of microbial colonies for each studied sample in colony-forming units per milliliter (CFU/mL) was performed by the same investigator (Figure 7). The number of colonies developed on the culture medium for the negative control samples has been quantified as 10^5^ CFU/mL. The results obtained in this regard following each type of treatment are reported in the related subsection.

### 2.3. Scanning Electron Microscopy (SEM)

SEM analyses were conducted using a high-vacuum FEI Quanta 250 system (Thermo Scientific™ Quanta™, Hillsboro, OR, USA) equipped with a secondary Everhard–Thomley electron detector. Operating parameters such as pressure and distance were adjusted as required for optimal imaging. Procedural steps included mounting on a conductive copper holder stub with adhesive carbon wafers on both sides. Samples were inserted into the SEM system at suitable magnification levels, ensuring direct exposure of the target area to the scanning electron beam with the guidance of a binocular microscope. Sample alignment on the stub prevented tilting during microscopic observation.

**Figure 8:**
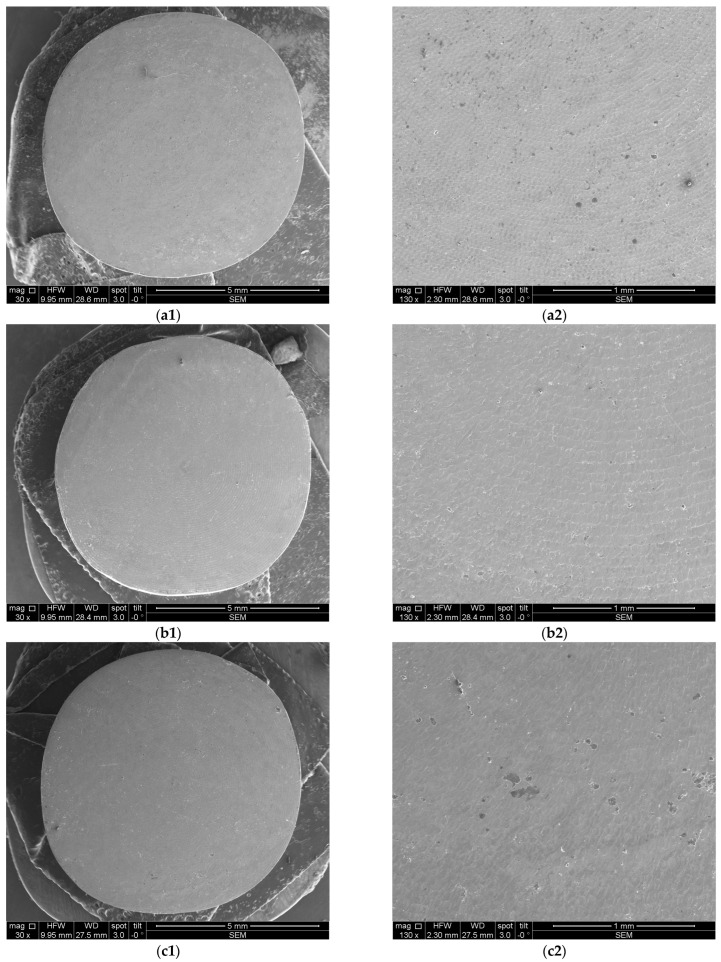

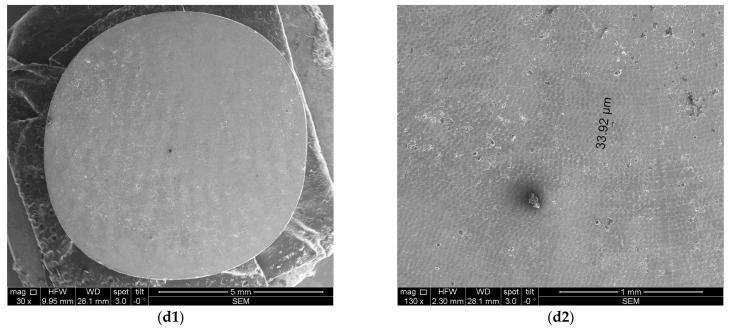
SEM images of Ti samples presented as examples from each study group: (**a**) CV, (**b**) Er:YAG QSP mode, (**c**) Er:YAG SSP mode, and (**d**) PDT and Er:YAG for a 30× magnification in column (1), as well as for a 130× magnification in column (2).

**Figure 9:**
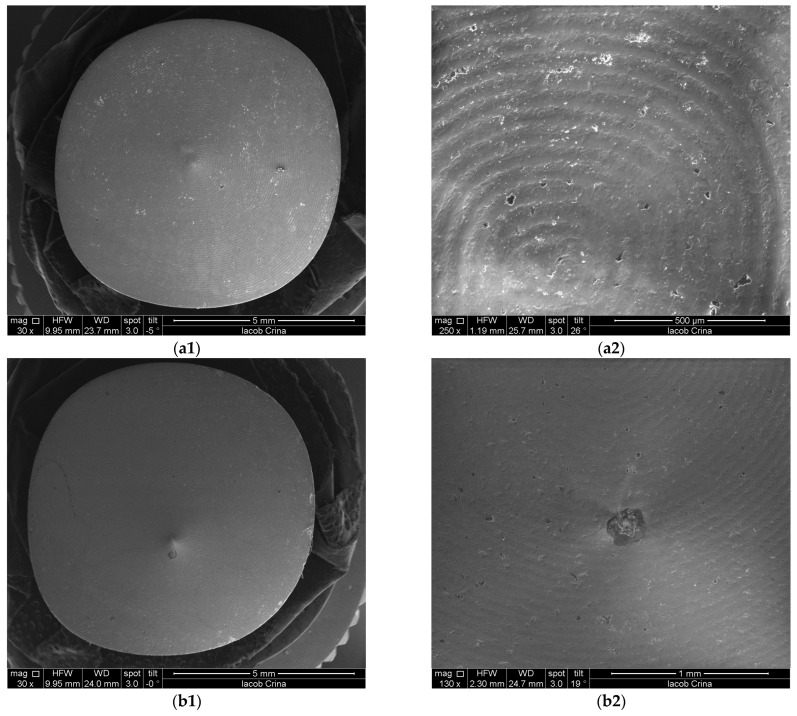

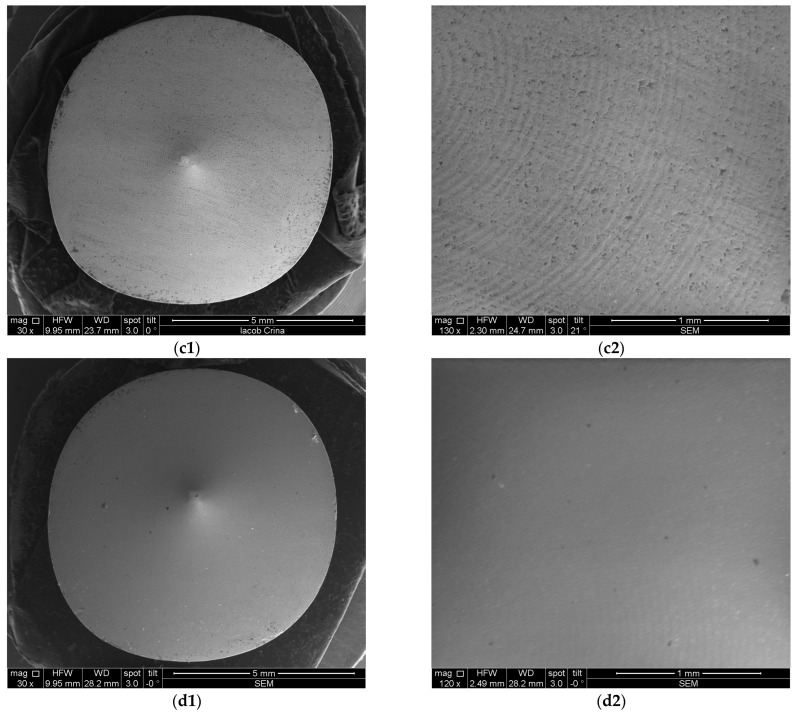
SEM images from Zr samples presented as examples from each study group: (**a**) CV, (**b**) Er:YAG QSP mode, (**c**) Er:YAG SSP mode, and (**d**) PDT and Er:YAG for a 30× magnification in column (1), as well as for a 130× magnification in column (2).

Figure 8 and Figure 9 depict representative examples of SEM images of the treated surfaces of the Ti and Zr samples, respectively. These images showcase the surface morphology, granularity, and microstructural details following specific treatment procedures. Notably, the SEM analysis required a gold coating to enhance surface conductivity and minimize charging effects during imaging.

### 2.4. Optical Coherence Tomography (OCT)

**Figure 10:**
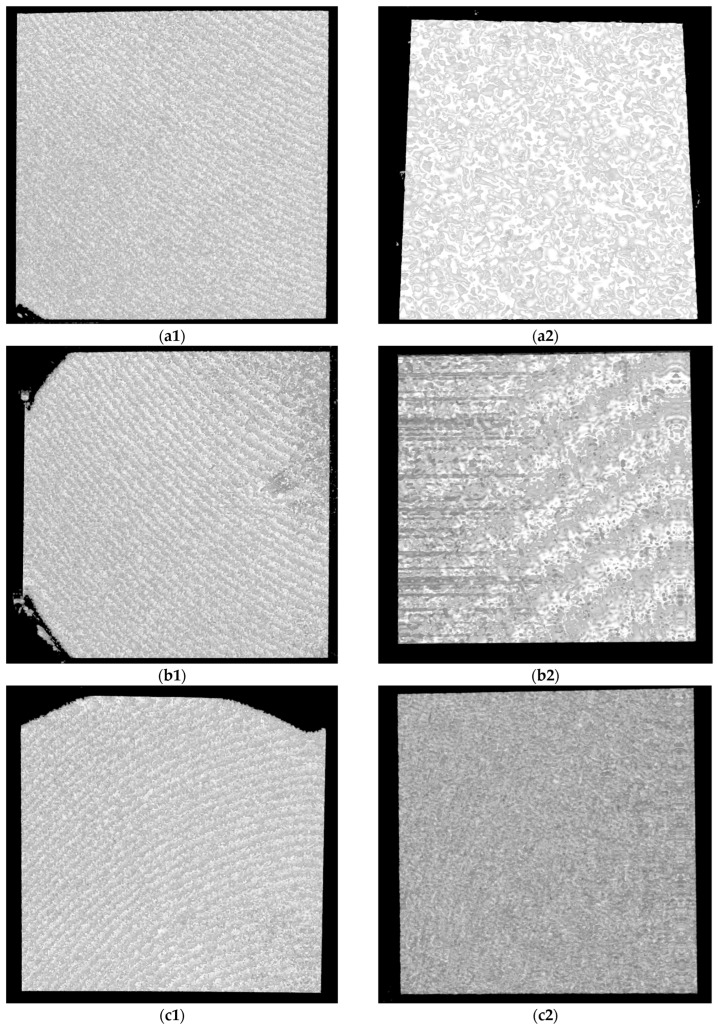

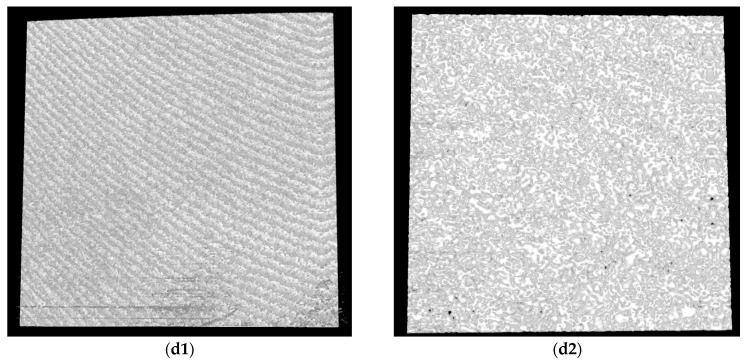
OCT images for Ti samples presented as examples from each study group: (**a**) CV, (**b**) Er:YAG QSP mode, (**c**) Er:YAG SSP mode, and (**d**) PDT and Er:YAG for the 5 × 5 mm investigated area in column (1), as well as for the 3 × 3 mm investigated area in column (2).

**Figure 11:**
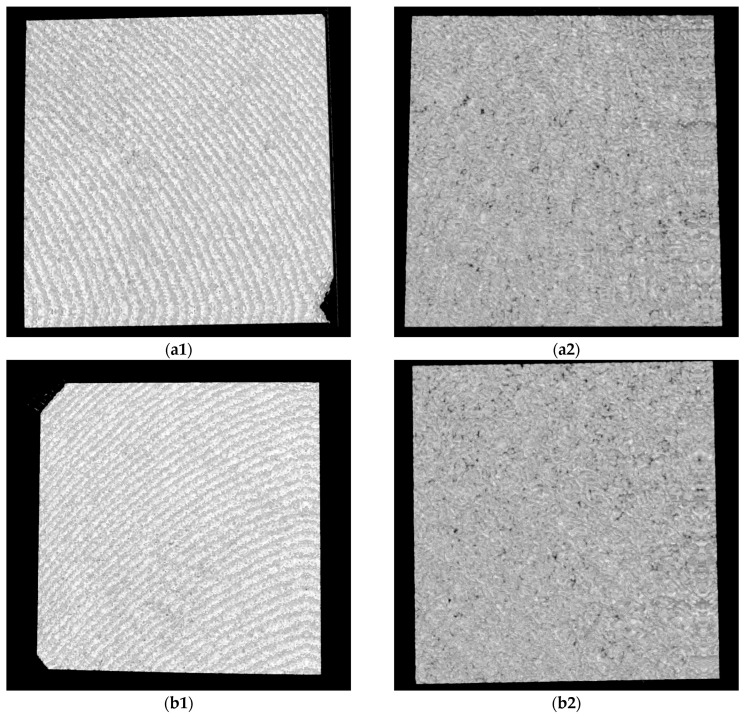

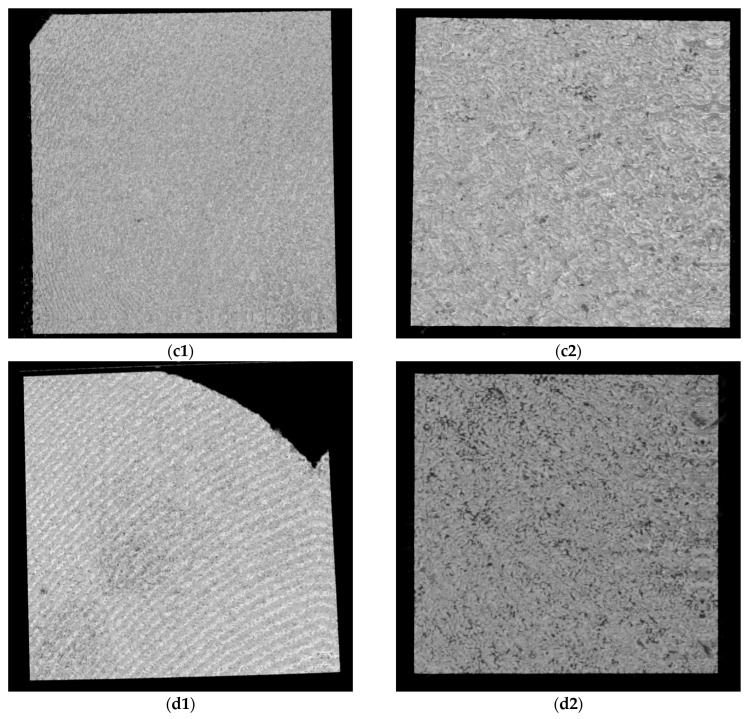
OCT images for Zr samples presented as examples from each study group: (**a**) CV, (**b**) Er:YAG QSP mode, (**c**) Er:YAG SSP mode, and (**d**) PDT and Er:YAG for the 5 × 5 mm investigated area in column (1), as well as for the 3 × 3 mm investigated area in column (2).

For OCT assessments, a custom-designed in-house developed SS-OCT system was employed, utilizing a 50 kHz swept source (i.e., a broadband laser source scanned in frequency (Exalos AG, Zürich, Switzerland)) with a central light wavelength of 1310 nm. Signal digitization from the output and control of the 2D galvanometer laser scanner of the system (employed in raster scanning) were managed using National Instruments hardware, including PXI5124 and PCI 6110 boards, as detailed in our studies [23,24,25]. An axial resolution of 15 μm in air characterizes the system. While the SEM investigations required a gold coating process, for OCT, no special preparation of samples was necessary before the investigations. This is an important advantage of the OCT method because, for this type of research, it may be essential to avoid altering the samples. Figure 10 and Figure 11 present examples of OCT images of the investigated surfaces.

### 2.5. SEM and OCT Image and Data Processing

**Figure 12:**
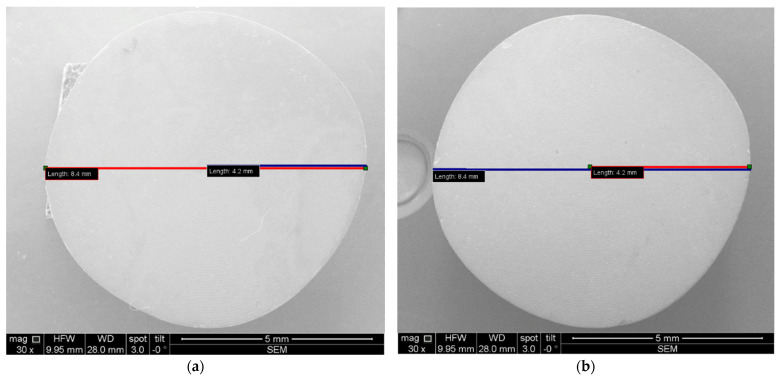
SEM images, where diameters and radiuses of two samples were measured for (**a**) Ti and (**b**) Zr.

**Figure 13:**
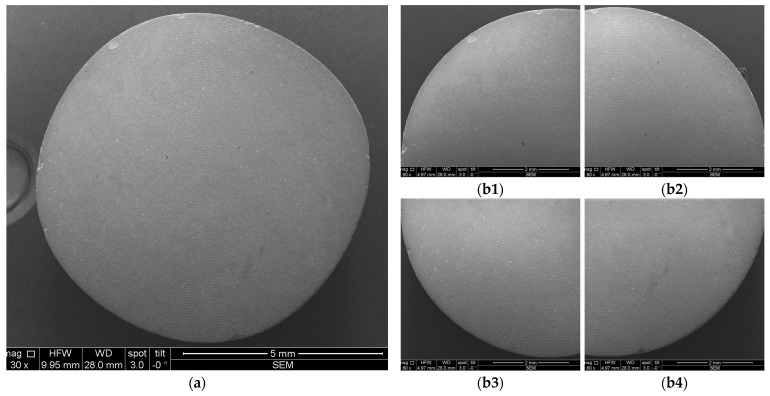
Example of SEM images acquired for measurements: (**a**) entire sample; (**b1**–**b4**) quadrants of the same sample.

Furthermore, the measurement protocol utilized throughout this study is presented. Notably, all images covering both OCT and SEM representations were imported and subjected to analyses using IC Measure (The Imaging Source Europe GmbH, Bremen, Germany). This versatile software facilitated precise measurements of parameters within the images, which benefited from the known sizes of both OCT and SEM images. The calibration process is shown for Ti and Zr samples in Figure 12 and Figure 13 for examples of obtained images.

Each sample was divided into four quadrants for SEM investigations and in two halves for OCT investigations in order to ensure a comprehensive coverage and analysis of each sample. Further on, the affected areas for quadrants (for SEM images) and for halves (for OCT images) were evaluated with the mentioned program. This allowed for statistical analyses of the results, as presented in detail in Section 3.

### 2.6. Descriptive and Inferential Statistics

Statistical analyses were conducted using IBM SPSS Statistics, Version 26.0, focusing on various parameters such as surface roughness and biofilm characteristics among the control, conventional, and laser-treated groups. The initial step involved verifying the normality of biofilm quantitative data, followed by comparing these parameters across groups through methods such as Fisher 95% Individual Confidence Intervals and Hsu’s Multiple Comparisons with the Best (MCB), specifically analyzing the Er:YAG group’s impact between Ti and Zr materials. Any *p*-value below 0.05 was deemed to indicate a statistically significant difference between groups, guiding the inferential assessment of the efficacy of each decontamination method. The Mann–Whitney U test was employed to determine if there is a significant difference between the two sets of independent measurements, specifically those obtained using OCT vs. SEM. The Kruskal–Wallis test was applied in order to investigate potential variations across study groups, specifically conventional, Er:YAG, and combined PDT and Er:YAG treatments.

## 3. Results

### 3.1. Microbiological Assessment

**Figure 14:**
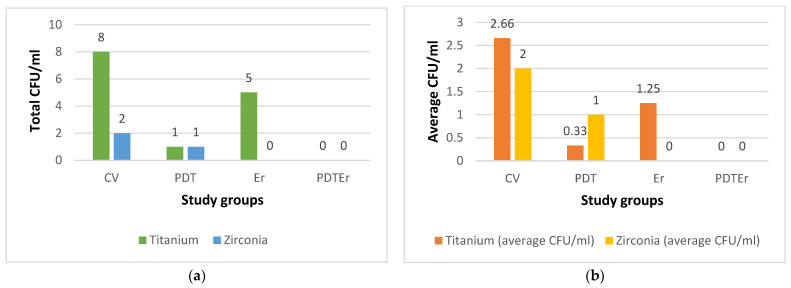
Results of the assessment of microbial colonies for each of the study groups (i.e., CV, PDT, Er, and PDTEr), quantified in colony-forming units per milliliter (CFU/mL): (**a**) total CFU/mL in each treatment group; (**b**) average CFU/mL for each treatment group, the latter calculated as the ratio between the total CFU/mL for each group (indicated on top of the bars in (**a**)) and the number of considered samples.

Quantitative analysis of microbial colonization was conducted by measuring colony-forming units per ml (CFU/mL) for each specimen. The results, i.e., CFU values for all groups, are shown in Figure 14. The aggregated data suggested a substantial post-treatment reduction in the microbial load compared to the initial values of 10^5^ CFU/mL, which characterized the samples of the negative control group. To point out the difference from Figure 14a to Figure 14b, we must stress that for groups CV and PDT, one of the four Ti samples was considered contaminated and was discarded; hence, the average values of 8/3 = 2.66 and 1/3 = 0.33 CFU.ml for these groups (and Ti samples). For Zr samples, we obtained 2 CFU/mL for a single CV sample (and 0 for the other three samples) and 1 CFU/mL for a single PDT sample (and 0 for the other three samples); hence, the obtained average in Figure 14b. For Ti samples of the Er group, the average is 5/4 = 1.25.

Statistically, the interventional arms, comprising PDT and the combination of PDT with Er:YAG laser (PDTEr), exhibited a marked decrease in CFU values compared to the negative control group, with t-values ranging from −2.33 to −2.67 and with all the *p*-values falling below the 0.05 threshold, therefore denoting statistical significance. Comparative evaluation within the interventional cohorts revealed that Ti substrates sustained a higher CFU count than Zr counterparts, underscoring a material-dependent differentiation in microbial reduction.

The results presented in Table 1 illustrate a statistically significant reduction in the viable bacterial counts across all decontamination study groups compared to the negative control group (C), with *p*-values smaller than 0.05 for both Ti and Zr surfaces. Specifically, the mean differences in bacterial reduction percentages were markedly high, with all study groups (i.e., Group CV, Group PDT, Group Er, and Group PDTEr) demonstrating near-complete eradication of microbial colonies when compared to Group C.

Moreover, when comparing the efficacy of PDT and Er:YAG laser treatments directly against the conventional therapy (CV), notable improvements were observed. Thus, the PDT group showed a mean difference of −2.33% (*p*-value = 0.040), and the PDTEr group (combining PDT and Er:YAG) showed a mean difference of −2.67% (*p*-value = 0.010). This indicates that the combined therapy approach was more effective in reducing microbial colonies on both Ti and Zr discs. When compared directly to PDT, the Er group presented an increase in the mean percentage, indicating that PDT was more efficient on both surfaces compared to using Er:YAG alone.

The analysis of Fisher 95% confidence intervals and Hsu’s MCB revealed a nuanced understanding of microbial colonization reduction on dental implant surfaces. Fisher’s analysis indicated no significant differences between PDT and its combination with ER:YAG laser treatment (PDT and Er:YAG), with confidence intervals for PDT ranging from −0.6113 to 1.1113 and for PDT and Er:YAG from −0.8613 to 0.8613; this suggested a comparable effectiveness in microbial reduction. Conversely, Hsu’s MCB underscored a significant material-dependent variance, with Zr surfaces exhibiting a substantial reduction in CFU, evidenced by a lower confidence interval range (i.e., −2.4726 to 0.0000) compared to Ti (i.e., 0.0000 to 2.4726). These findings emphasized the material-specific response to decontamination treatments and highlighted the superior performance of Zr in microbial reduction, advocating for its consideration in dental implantology in order to optimize clinical outcomes, as presented in Table 2.

### 3.2. Temperature Variations

Initial and maximum temperatures were recorded for samples under PDT or Er:YAG laser treatments, utilizing a FLIR T640 infrared thermographic camera. For the PDT group, the Ti samples showed an average initial temperature of 23.58 °C and a maximum temperature of 24.88 °C during the treatment. The Zr samples in the same group exhibited initial temperatures averaging 21.35 °C and maximum temperatures averaging 23.8 °C. The Er:YAG group demonstrated slightly lower initial temperatures for Ti at 21.18 °C and for Zr at 20.75 °C, with maximum temperatures reaching 21.45 °C for Ti and 22.43 °C for Zr during treatment, as presented in Table 3.

These measurements indicated minimal temperature increases, maintaining a tight range between 21 °C and 25 °C, with modest variation. This suggests a negligible risk for thermal degradation of the implant surface materials. The stable thermal environment ensured by Er:YAG laser treatments highlights the technique’s efficacy in preserving the integrity of both Ti and Zr surfaces without causing significant thermal-induced material changes. Further statistical analysis using the Tukey Method to group mean temperature responses concluded no significant differences among the groups, categorizing them all under the same group (A). Specifically, the mean temperatures were 23.57 °C for PDT-Ti, 21.35 °C for PDT-Zr, 21.17 °C for Er:YAG-Ti and 20.75 °C for Er:YAG-Zr. This uniformity in thermal response across the various treatment groups and materials emphasizes the consistency and safety of the Er:YAG laser application in dental treatments by ensuring a minimal thermal impact on both Ti and Zr surfaces.

### 3.3. Imagistic Evaluations Using SEM and OCT

**Figure 15:**
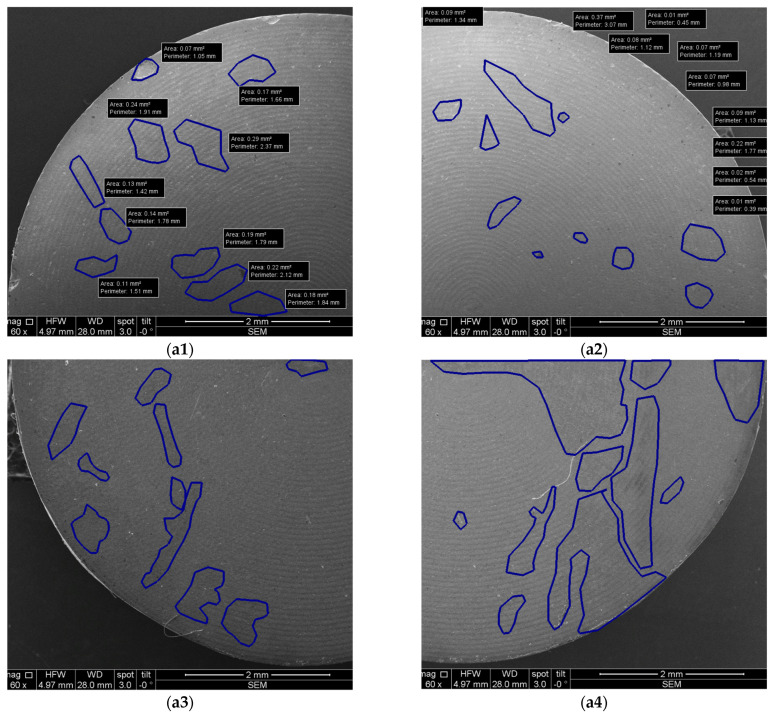
Example of SEM images with measurements: (**a1**–**a4**) quadrants of a sample.

**Figure 16:**
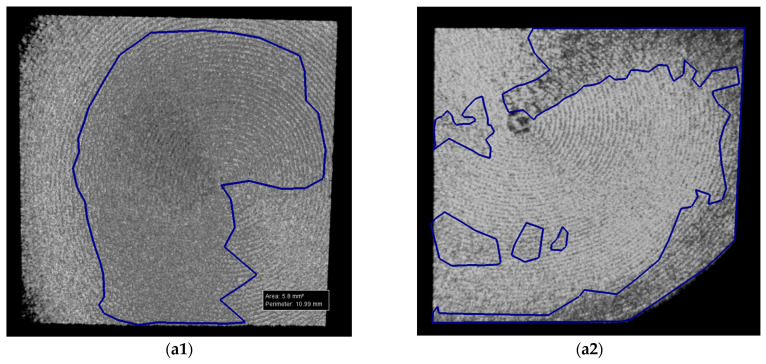
Example of OCT images with measurements: (**a1**,**a2**) halves of a sample.

Both Ti and Zr samples underwent imagistic evaluations using SEM and OCT in order to analyze surface morphological features and micro-structural changes for each study group (i.e., after each type of treatment) in order to assess the eventual surface degradation induced by each type of decontamination process. Using the corresponding systems previously described, both SEM and OCT images were obtained. An example of the processing of such images is presented in Figure 15 for SEM and in Figure 16 for OCT.

The analyses performed using both OCT and SEM are presented in Table 4 regarding the effects of various treatments on Ti samples. The numbering of the samples corresponds to Table 3, with the notation of samples 1 to 4 for the CV group.

The results reveal insightful data on surface modifications. Thus, in the conventional group (CV) and in those treated with Er:YAG laser in SSP or QSP modes, along with the combined PDT and Er:YAG treatment, OCT and SEM documented distinct levels of surface area alterations. Specifically, OCT results showed the total affected area ranging from 1.84 mm^2^ to 5.22 mm^2^ for CV, with SEM findings extending from 1.74 mm^2^ to 9.96 mm^2^. This highlights SEM’s higher sensitivity in detecting finer surface changes. Particularly notable was the SSP mode treated samples where OCT and SEM measurements, although generally consistent, indicated a variation in the treatment impact, with significant discrepancies in some samples, for example, 13.85 mm^2^ by OCT vs. 15.91 mm^2^ by SEM.

Moreover, the combination of PDT and Er:YAG treatment indicated an augmented surface area modification, with OCT capturing changes from 0.7 mm^2^ to 4.07 mm^2^ and SEM findings ranging from 1.26 mm^2^ to 16.51 mm^2^. This suggests a synergistic effect of combined treatments in modifying surface characteristics, potentially improving microbial decontamination efficiency.

Table 5 is focused on Zr samples, and it provides a comparative analysis between OCT and SEM in the ascertainment of surface area changes following various treatments. For the conventional group, OCT measurements depicted the total affected area as ranging from 0.24 mm^2^ to 2.31 mm^2^, while SEM analyses indicated a slightly wider impact, from 1.19 mm^2^ to 5.66 mm^2^. The Er:YAG treatment in SSP mode showed OCT-determined affected areas between 1.54 mm^2^ and 2.54 mm^2^, closely mirrored by SEM, which recorded changes between 0.97 mm^2^ and 3.65 mm^2^, suggesting a moderate yet significant alteration in surface integrity.

Moreover, the combined PDT and Er:YAG approach pointed out a notable increase in surface area changes, with OCT capturing alterations from 1.47 mm^2^ to 4.19 mm^2^, while SEM documented a more extensive range, from 0.88 mm^2^ to 11.67 mm^2^. The QSP mode of Er:YAG treatment presented the smallest impact, with affected areas documented by OCT and SEM closely aligned, thus highlighting a conservative approach to surface alteration.

The Mann–Whitney U test was employed to determine if there is a significant difference between the two sets of independent measurements, specifically those obtained using OCT vs. SEM, as detailed in Table 6. The test yielded a U value of 928.5 and a Z-score of 0.32547, resulting in a *p*-value of 0.3707, which suggests that there was no statistically significant difference between the OCT and SEM methods in evaluating the characteristics of the areas of the affected surfaces.

Table 6 highlights the comparative analysis of the surface characteristics as measured using OCT and SEM, with the mean surface area affected recorded as 4.0064 mm^2^ for OCT and slightly less, at 3.9889 mm^2^, for SEM. The mean absolute deviation and SD for OCT measurements indicated slight variations in the spread of data. Furthermore, the SE of the mean (i.e., 0.3089 for OCT and 0.3218 for SEM) demonstrated the close alignment in terms of precision between the two evaluation methods.

The Kruskal–Wallis test was applied in order to investigate potential variations across study groups, specifically conventional, Er:YAG, and combined PDT and Er:YAG treatments. This statistical analysis yielded an H statistic of 0.1087 and a *p*-value = 0.74167, notably exceeding the significance threshold of 0.05. Mean rank scores were calculated as 19.97 for CV, 23.84 for Er:YAG, and 29.69 for PDT and Er:YAG. In these pairwise comparisons among these groups, none revealed *p*-values below the 0.05 significance level, reinforcing the conclusion that there are no significant differences between specific pairs of groups.

Table 7 presents comparative measurements of the impact on sample surfaces among the CV, Er:YAG, and PDT and Er:YAG groups.

They indicate mean surface impact measurements of 3.55 mm^2^ for CV, 4.25 mm^2^ for Er:YAG, and 5.31 mm^2^ for the combination treatment. Despite these numeric differences, the Kruskal–Wallis test results, alongside an effect size (*η*^2^ = 0.042), suggested no statistically significant disparities among these treatments regarding their impact on sample surfaces. Therefore, based on the provided data and subsequent analysis, there is no compelling evidence of variations in the dependent variable across these treatment groups, emphasizing a uniform impact of these therapeutic modalities on sample surfaces.

The analysis that compares Ti and Zr measurements leveraged the Mann–Whitney U test to discern any statistical differences between the two material groups, as detailed in Table 8. The examination of mean values presented for Ti and Zr (i.e., 9.3795 mm^2^ and 6.5505 mm^2^, respectively), along with Mean Absolute Deviations (i.e., 3.1831 mm^2^ for Ti and 2.0324 mm^2^ for Zr) highlighted the variances in measurements between the two materials.

The Mann–Whitney U test’s results (featuring a statistic H of 5.7322 with degrees of freedom (df) = 1), yielding a *p*-value of 0.01666) surpass the significance threshold of 0.05, thus revealing a statistically significant difference between the Ti and Zr groups. This outcome necessitates the rejection of the null hypothesis (H0), which posited no significant difference between the measurements for Ti and Zr samples. Instead, the alternative hypothesis (H1), suggesting a notable variance between the two types of materials, is accepted. Consequently, this statistical evidence leads to the conclusion that Zr surfaces are less impacted by treatments compared to Ti, thus showcasing a distinct material response to similar treatment conditions.

## 4. Discussion

### 4.1. Dental Implants Materials

Ti and its alloys have been suggested as standard materials for dental implants because of their proven biocompatibility and superior corrosion resistance properties compared to stainless steel and cobalt–chromium alloys. The biocompatibility of Ti is based on using a thin layer of passive oxide (i.e., TiO_2_) on the surface; this layer is formed when Ti is exposed to air or to body fluids [29]. Also, the high strength and the low modulus of elasticity of Ti compared to other metals allow for withstanding the forces that are applied to Ti-based parts.

The covering of endo-osseous Ti dental implants with the oxide layer favors the adhesion of biomolecules, cell binding, and, therefore, osseointegration. In contrast, the contamination of the implant surface reduces the surface tension, and this aspect further affects the adhesion capacity [29,30]. The oxide layer prevents ion exchange with the external environment, acting as a dense protection. This aspect may explain the biocompatibility and corrosion resistance of the material. Also, it has been suggested that this layer is responsible for the initiation and enhancement of osseointegration [31].

In addition, biological tissues exhibit a high level of compatibility with pure Ti. However, it is uncertain whether the presence of other materials affects the biological response of the anatomical sites where implants were inserted [32].

Zr-based implants are a possible substitute for Ti and related alloys because they offer an equivalent osseointegration and better biocompatibility, as well as soft tissue response [31]. Numerous microbiological and in vivo studies have shown that there are fewer cocci and rods surrounding Zr implants than around Ti implants (with higher levels of *Streptococcus mutans* and with lower levels of *Streptococcus sanguis*) [32]. The development of peri-implant bone resorption and peri-implant soft inflammation is prevented by these bioinert characteristics, which improve peri-integration around Zr implants [33]. In addition to these beneficial characteristics, Zr surfaces tend to accumulate less tooth plaque compared to Ti ones, without showing differences in the bone-implant contact and osseointegration process when compared to Ti implants [34]. These studies on the bioinert characteristics of Zr offer a possible explanation for the superior results obtained in our study in the decontamination of Zr discs compared to Ti ones.

### 4.2. Pathogen’s Decontamination

Zhuang et al. compared the prevalence and levels of six bacterial pathogens within the subgingival/submucosal microbiota at teeth versus implants with various clinical conditions [35]. The most commonly detected species were *Staphylococcus aureus* and *Fusobacterium nucleatum*, while *Aggregatibacter actinomycetemcomitans* and *Prevotella intermedia* had the lowest detection frequency. The detection frequencies of diseased teeth or implant sites for each of the six targeted species were either equal to or higher compared to the frequencies identified in the corresponding healthy sites [36].

In a systematic review by Gazil et al., the authors of the eight included studies agreed that PI has a different microbiota than periodontitis and is a separate pathological entity [16]. Six included studies examined the diversity of the microbiota based on ecological niches, but no consensus emerged because two studies found no difference in diversity [37,38], while two studies found that healthy sites had a richer microbiota than those associated with PI [24,39]. Two other studies found more microbiological diversity associated with PI [23,30]. These findings are consistent with systematic reviews conducted by other authors [40]. These scientific results encouraged us to use *S. aureus* as the species of choice in our study.

The various decontamination techniques have both advantages and drawbacks, the latter including mechanical- or laser therapy-caused surface damage, as well as chemical treatment failing to remove all contaminants [41]. Plastic curettes, ultrasonic scalers, and airflow abrasion are the most common decontamination strategies for implants. However, conventional strategies have limited effectiveness due to the implant surface’s microscopic porosities [42].

#### 4.2.1. Conventional Decontamination Methods

This step can be performed through mechanical and chemical techniques. Several in vitro studies have demonstrated the ability of chemical agents to decontaminate and interact with microbes [43]. In the present study, we did not utilize non-surgical mechanical debridement for the control group as it proved ineffective in treating PI [44]. Instead, we employed a combination of mechanical and chemical treatments for the control group (C) based on the available evidence.

An initial phase of non-surgical debridement and decontamination may reduce inflammation and can even act as an independent treatment in moderate cases or to precondition soft tissues before surgery [45]. Implant surface debridement instruments have been proposed in this respect. The most common are curettes, ultrasonics, and abrasives. A recent in vitro implant debridement study found that air powder devices clean better than curettes or ultrasonic instruments [30]. Even so, modern treatments have shown limited medical benefits and little microbiological improvement six months later [46]. Saline solution, delmopinol, chlorhexidine, tetracycline, CHX minocycline, citric acid, hydrogen peroxide, EDTA, or 35% phosphoric acid gel have been applied for chemical decontamination. However, no implant surface decontamination method has proven completely effective. Also, there is no consensus on the best PI recovery treatments. Supplemental systemic antibiotics have little effect on treatment success. On the other hand, widespread antibiotic use may cause side effects and select antibiotic-resistant bacteria [38].

Chlorhexidine digluconate targets gram-positive, gram-negative, and yeast bacteria; therefore, it is often utilized to reduce PI bacteria. Antibiotics leak and eliminate bacteria by altering their cell membranes [47]. Concentration and exposure time determine CHX cellular toxicity. The osteoblastic phenotype does not change after 1 min. of 0.2% CHX and 30 s of 1% CHX. Also, CHX inhibits cell growth and collagen production. Such negative effects may explain why bacteria decreased and tissue healing did not improve [48]. Positive results in this respect include the following: (i) medical subgingival irrigation with 0.2% CHX and mechanical debridement reduced bacteria and improved probing depth for up to 3 months [49]; (ii) against *Streptococcus sanguinis* and *Candida albicans*, CHX (0.2%) for 60 s worked in vitro [50,51]; (iii) using a cotton pad soaked in 0.12% to gently press on the implant surface for 1 min. reduced *Porphyromonas gingivalis* endotoxin by up to 92.9% [38]; (iv) a study found that 0.2% CHX gel after mechanical cleaning and 1 mg of minocycline microspheres in the peri-implant defect reduced microbiota in 48% of cases [49].

The use of air-powder (AP) abrasion yields favorable outcomes. AP abrasion is a dental technique that uses compressed air to remove biofilm or extrinsic stains from teeth. The process involves the use of sodium bicarbonate or amino acids such as glycine [52]. Researchers have demonstrated that a combination of water, air, and powder at a pressure of 65 to 100 pounds per square inch (psi) effectively removes contaminants from implant surfaces in laboratory and living organism experiments [53]. In an in vitro study conducted by Tastepe et al. [42], sandblasting demonstrated superior performance compared to the topical application of citric acid, tetracycline, HCl, CHX, hydrogen peroxide, chloramine T, distilled water, and plastic ultrasonic scaling tools. According to another laboratory study, AP abrasion was able to eliminate bacterial endotoxin from 98.5% of five Ti-machined surfaces, 84.2% of plasma-sprayed surfaces, and 88.8% of HA-coated surfaces [54]. Maximo et al. demonstrated, in laboratory conditions, that using Teflon curettes and AP abrasion for surgical access and implant debridement resulted in improved clinical parameters and a decrease in periodontal pathogens [55].

#### 4.2.2. Photodynamic Therapy (PDT)

Laser dentistry has transformed both the research field and modern clinical dentistry. Today, a variety of lasers are used in dental procedures to reduce bleeding, inflammation, and pain. The most common lasers include diode and Er:YAG, CO_2_ lasers, and PDTs [56,57]. They can be used for both nonsurgical and surgical procedures [58].

In our study, PDT alone was ineffective in completely eliminating *S. aureus* from all Ti surfaces, implying that for a more complex bacterial load, PDT alone may not be enough as a sole Ti surface disinfection modality [59]. This is consistent with previous research, which found that using PDT with different photosensitizers reduced bacterial load on Ti surfaces but did not completely eliminate it [15].

Our previous clinical study on PDT in periodontitis patients discovered that it reduces the number of pathogenic bacteria in periodontal pockets [60]. Given that the bacterial biofilm associated with PI is similar to that involved in the pathology of periodontitis, PDT may be an alternative solution for decontaminating implant surfaces because it does not damage the surface within a specific wavelength range and has an antibacterial effect [61].

Using both brushing and PDT effectively decreases the presence of *S. aureus* on both smooth and uneven Ti surfaces [62]. PDT is not effective for treating deep bacterial infiltrations. PDT enhances periodontal inflammatory indices, as pointed out by certain research [27]. PDT requires the presence of a photosensitizer, the application of light, and the availability of oxygen. The treatment exhibits cytotoxic effects on mitochondria, lysosomes, and cell membranes. Insufficient power density prevents the laser beam from penetrating deeper because of the absorption by the chromophore at certain wavelengths [63].

The use of PDT may be more convenient than mechanical instrumentation because of its straightforwardness. PDT protocols employ low-level laser therapy (LLLT) to prevent thermal harm, rendering decontamination inadequate. There is a growing preference among dental professionals for non-surgical periodontal therapy, which involves the use of a 980 nm laser and hydrogen peroxide [64]. In comparison to photo-activated PDT, utilizing a high-frequency diode laser (980 nm) and hydrogen peroxide irrigation of periodontal pockets resulted in enhanced tissue absorption and biofilm penetration [65].

#### 4.2.3. Er:YAG Laser

The Er:YAG laser has been proposed as ideally suited for the decontamination of implants because it is primarily absorbed by water. This characteristic results in bacterial vaporization and minimal implant surface damage [66,67]. The majority of studies in the literature have been using output ranges from 80 to 120 mJ/pulse [15]. Yamamoto and Toshijichiro et al. suggested that an energy output of less than 100 mJ/pulse probably effectively disinfected the surface [68]. Because of the low absorbance of the Er:YAG laser in Ti, adverse thermal effects such as carbonization, melting, and heat injuries to adjacent tissues have not been observed [69].

Matys et al. found in a series of studies that increasing the temperature of bone tissue by 10 °C for approximately 1 min causes permanent changes in the bone structure. Therefore, a tissue temperature gradient (ΔTa) below 10 °C should be regarded as optimal and safe [70]. The implant temperature gradient depends on the differences in the physical and chemical properties of the Ti grades, particularly thermal conductivity, which is almost three times lower in grade V than in grade IV Ti [71].

The possible temperature variations during the use of the Er:Yag laser were also of interest to us in our study monitoring these increases during treatments.

Numerous reports have analyzed the relationship between increases in implant surface temperature and the amount of laser energy. However, to our knowledge, no comparative study has been conducted on how much diode and Er:YAG lasers at different energy settings increase implant temperature, considering the width and chemical composition of implants. In their comparative analysis of increases in soft tissue temperature, Merigo et al. concluded that the effect of Er:YAG lasers resulted in a smaller rise in tissue temperature compared to the effect of diode or CO_2_ lasers [72]. Therefore, this was one of the aims of the present study.

Previous studies have shown that the effectiveness of the Er:YAG laser in removing contaminants ranges from 59% (80 mJ/pulse, 5 Hz) to 99.94% (120 mJ/pulse, 10 Hz) in a dose-dependent manner. However, high-energy Er:YAG laser irradiation should be avoided in order to prevent additional chemical contamination and minimize mechanical and thermal damage to the Ti surface microstructures [73]. Also, Fornaini et al. emphasized that the values of the mean increase in temperature on the implant surface were, for the diode laser, 12.67 °C; for the Nd:YAG laser, 16.86 °C; for the Er:YAG laser, 1.78 °C; for the KTP laser, 7.7 °C. It can be seen that an Er:YAG laser causes a smaller increase in implant temperature compared to a diode laser [74].

A study found that the radiation produced by lasers is poorly absorbed by Ti at specific wavelengths. Therefore, it does not significantly increase the temperature of the implant. However, an in vitro study with Er:YAG found that Ti discs showed changes after 10 s of irradiation at 300 mJ/10 Hz; they were characterized by tip melting. Cracks were formed on polished Ti surfaces after exposure to an irradiation of 500 mJ/10 Hz. These surface changes can be associated with a thermal effect produced by a minimal thermal relaxation between pulses [75].

In Matsuyama’s experiments, it was found that the Er:YAG laser irradiation at a high output energy causes a surface change of Ti. Thus, distinct morphological and color changes occurred on the Ti surface when irradiated by Er:YAG laser at 100 or 200 mJ/pulse. However, few morphological and color changes were observed on the Ti surface at 30 or 50 mJ/pulse [76]. These findings mean that there is a threshold of energy output for the occurrence of Ti surface alternation after the Er:YAG laser irradiation [77]. Our group has carried out previous research in which we investigated the interaction between Er:YAG laser radiation and the surface of Ti implants, as well as their osseointegration. We concluded that a conventional bur preparation produced more detritus and blood cells than a laser preparation; this may be detrimental to the osseointegration process. The use of a QSP mode showed more successful performances in terms of reducing heat impacts and producing well-defined cavities compared to using the SSP mode. Overall, findings indicate that a laser may be a beneficial instrument for creating an implant site for patients obtaining implant-supported restorations [78].

Renvert et al. found a limited and similar effect when comparing a YAG laser and an air abrasion device in treating clinical cases with PI. The results of the therapy of subjects with PI after 6 months were similar using an Er:YAG laser or air abrasion for the debridement of implants diagnosed with severe PI. Both methods resulted in a reduction of periodontal pocket depth, as well as of the frequency of suppuration and bleeding at implants with a diagnosis of PI. However, the overall clinical improvement was limited [79].

An in vitro study suggested that the laser wavelength does not change the roughness and morphology of both smooth and rough surface implants, except for minor damages caused by contact with the utilized tip [80]. Due to the impracticality of maintaining a consistent distance between the laser beam and the implant surface in real-world clinical settings, we employed an experienced clinician to estimate this distance in our current study. Moreover, in the majority of scientific protocols, it is customary to employ chemical techniques (such as CHX or hydrogen peroxide rinsing) as an additional measure [81]. Hence, in the current study, CHX rinsing was performed uniformly on all slices to replicate conditions within the mouth.

After PI disease therapy, it is necessary to make certain that soft tissue attachment is favorable to have a suitable mucosal seal. Pham et al. examined Zr discs and confirmed clear surface adjustments with the use of ultrasonic instrumentation and hand scalers, in contrast to the Er, Cr: YSGG laser crew and manage group; also, no distinction in the surface microroughness was seen. The Er, Cr: YSGG laser was capable of successfully ablating microorganisms from Zr discs; therefore, they were proved to be more effective compared to hand instrumentation. Fibroblast attachment on the surfaces of Zr discs indicates extra adherence when handled with Er, Cr: YSGG laser [82].

Contrary results were also found. Thus, in an in vitro experiment, Er:YAG was used to treat intraorally contaminated discs. Compared to the use of plastic curettes and an ultrasound system, it was shown that it can effectively remove supragingival biofilm, but it fails to restore the biocompatibility of the surfaces.

Romanos and Nentwig investigated the effectiveness of a CO_2_ laser in the decontamination of failed implants. After a mean follow-up of 27 months, almost complete bone regeneration has occurred in the peri-implant defects [83]. Also, on dog models, surface decontamination was performed with an Er:YAG using 62 mJ/20 Hz for approximately 1.5 min. The implants were re-implanted for 6 months after decontamination. New bone contact with the implant in the defect area was 69.7% compared to 39.4% obtained with the plastic curettes used in the control group. The authors stated that by using the laser, surface decontamination and removal of granulation tissue were achieved without visible macroscopic damage to the surface [84]. These findings are in accordance with the results of our study.

#### 4.2.4. Investigation Methods

A range of imaging and testing methods have been used to report biological outcomes with surface modification, including SEM, transmission electron microscopy, atomic force microscopy, micro-computed tomography, synchrotron-based X-ray tomographic microscopy, confocal microscopy, and OCT [85]. Spectroscopy techniques have been utilized as well [86]. For the present study, SEM and OCT were selected for the performed investigations.

Since its inception and introduction to the area of dental research, SEM has been utilized for a number of objectives pertaining to dental biomaterials and dental hard or soft tissues, including the evaluation of micro-cracks/gaps and deformities in biomaterials and dental hard tissues, details of surface topography, roughness and subsurface structures of various dental biomaterials or dental hard tissues, as well as differentiation of various dental hard tissues [87,88].

The study of soft and hard tissues (such as dentin, enamel, and bone), cell culture (e.g., mineralizing bone cells), and dental materials are included in dental research (the latter includes adhesives and composites, metals, and ceramics). SEM is vital to the characterization of such materials [86,87,88,89,90]. By scanning the surface of the material with an electron beam and by detecting the produced secondary or backscattered electrons, resolutions of 10 nm or less can be obtained, exposing the structural characteristics of the material. Such characteristics have a substantial effect on the success or failure of a biomaterial device, emphasizing the importance of precisely defining the biomaterial surface [87]. In SEM, an electron beam scans the surface of the sample to generate a spectrum of signals, the characteristics of which depend on several parameters, such as the energy of the electron beam and the nature of the sample. This is essential in dentistry, as dental tissues and dental materials tend to be white or bright in color, making the use of optical microscopy challenging [88,90].

OCT is an optical technique that allows for non-invasive, in-depth imaging of different structures [17,18,19,20,21,22,23,24,25], including for dental hard tissues and dental materials [91]. OCT employs near-infrared light with wavelengths between 800 and 1500 nm to generate micrometer resolution 2D images or 3D reconstructions of a sample [17,18,19,20,21,22,23,24,25]. In our study, we included a custom-designed, in-house-developed SS-OCT system, utilizing a broadband laser source scanned in frequency with a central wavelength of 1310 nm, as described in detail in [23,24,25,92,93]. OCT has been mostly used in the diagnosis of dental hard tissues and cariology in dentistry [93,94,95]. However, the application of OCT has risen significantly in other dental sectors as well, including dental materials and prosthodontics. The technique creates new opportunities by filling a void between high-resolution optical microscopy and medical ultrasound in terms of resolution and scanned area size.

From the point of view of imaging investigation methods, the present study demonstrated that the lower cost (but also lower resolution) OCT can replace the costlier (but also higher resolution) SEM. This is in line with our findings in previous studies focused on different biomedical and materials applications [23,24,25].

The present study included a range of treatments, including conventional (mechanical and chemical) decontamination methodologies, PDT, Er:YAG laser treatment (Er), and a combined PDT and Er:YAG treatment (PDTEr), alongside a negative control group (C) that received no treatment for decontaminating Ti and Zr surfaces. Thermal analysis, SEM, and OCT were utilized to assess the (possible) degradation of the surfaces of the samples.

Research into the mechanism of the considered therapies has not been the purpose of this study but rather their efficacy and effects in the decontamination process. Thus, a characterization of the PDT therapy may include, for example, measurements of reactive oxygen species (ROS) and reactive nitrogen species (RNS) [96,97]). This was a limitation of the present study. However, one must consider the challenges regarding the dynamics of ROS, as both ROS and RNS exhibit an increased reactivity, leading to short half-lives in biological environments. Future work may target such characterizations of different methodologies, as well as randomized clinical trials to assess microbial destruction by different laser-emitting modes and different photosensitizers.

Another important aspect concerns the complexity of the microbial flora in the oral biofilm. While the results of this study have been exemplified by using *S. aureus ATCC 25923*, they should also be verified for other bacterial species that are specific to the oral cavity. It is often preferable to test first a new research hypothesis in a small sample of subjects. This avoids the unnecessary expenditure of resources for establishing a link between a factor and a certain disorder when there might be no effect.

Nevertheless, the in vitro study is unable to capture the inherent complexity of organ systems and the internal environment of the human body. Thus, in vitro cell culture may fail to account for interactions between different body procedures and cellular biochemistry. Therefore, progress in this direction must consider future in vivo studies.

## 5. Conclusions

This in vitro study evaluated the efficacy of PDT, Er:YAG laser radiation, and their combination (PDTEr) against conventional methods for reducing *S. aureus* colonies on Ti and Zr surfaces. Findings demonstrated that PDT and PDTEr protocols were notably more effective than conventional treatments, with PDTEr achieving complete eradication of microbial colonies in certain cases. Temperature assessments during treatments indicated no significant changes, suggesting the safety of these modalities. Surface examination using OCT and SEM revealed consistent treatment impacts, with no substantial differences in surface alterations between these two evaluation methods. Thus, the study also demonstrated that OCT is able to replace the common imaging method, SEM, for the considered investigations. Overall, while PDTEr and PDT showed enhanced antibacterial efficacy, the resilience of Zr surfaces suggested a preference for its use in environments requiring stringent microbial control.

## Figures and Tables

**Table 1:** Comparison between Ti and Zr samples, study groups, and negative control groups.

Study Group	Comparing Group	Mean Difference (%)	SE of the Mean	*p*-Value
Ti				
Group CV	Group C	−99.9	0.67	<0.001
Group PDT	Group C	−99.9	0.67	<0.001
Group Er	Group C	−99.9	0.62	<0.001
Group PDTEr	Group C	−100	0.62	<0.001
Group PDT	Group CV	−2.33	0.72	0.040
Group PDTEr	Group CV	−2.67	0.67	0.010
Group Er	Group CV	−1.42	0.67	0.270
Group Er	Group PDT	0.92	0.67	0.660
Group PDTEr	Group PDT	−0.33	0.67	0.990
Group PDTEr	Group Er	−1.20	0.62	0.320
Zr				
Group CV	Group C	−99.9	0.35	<0.001
Group PDT	Group C	−99.9	0.35	<0.001
Group Er	Group C	−100	0.35	<0.001
Group PDTEr	Group C	−100	0.35	<0.001
Group PDT	Group CV	−0.25	0.35	1
Group PDTEr	Group CV	−0.50	0.35	0.630
Group Er	Group CV	−0.50	0.35	0.630
Group Er	Group PDT	−0.25	0.35	0.950
Group PDTEr	Group PDT	−0.25	0.35	0.950
Group PDTEr	Group Er	0.00	0.35	1

Notation: SE—Standard Error, defined as the measure of the statistical accuracy of an estimate equal to the standard deviation of the theoretical distribution of a large population of such estimates.

**Table 2:** Comparative analysis of confidence intervals and microbial colonization on dental implant surfaces.

Test	Group	Lower	Center	Upper
Fisher 95% CI	PDT	−0.6113	0.2500	1.1113
Fisher 95% CI	PDT and Er:YAG	−0.8613	0.0000	0.8613
Hsu’s MCB	Ti	0.0000	1.2500	2.4726
Hsu’s MCB	Zr	−2.4726	−1.2500	0.0000

Notation: CI—confidence interval.

**Table 3:** Measurement of temperature levels during treatments.

Ti Study Groups	Sample	Initial Temperature t (°C)			Maximum Temperature t_max_ (°C) during Treatment		
		Value	Average	SD	Value	Average	SD
PDT	5	25.2	23.58	1.35	25.8	24.88	1.36
	6	22			23.6		
	7	22.5			23.5		
	8	24.6			26.6		
Er:YAG	9	22.8	21.18	1.05	21.3	21.45	0.23
	10	21.2			21.6		
	11	20.8			21.2		
	12	19.9			21.7		
**Zr study groups**							
PDT	5	21.5	21.35	0.26	23.6	23.8	1.10
	6	21.5			24.7		
	7	21.5			22.6		
	8	20.9			22.2		
Er:YAG	9	17.8	20.75	1.72	22.8	22.43	0.73
	10	21.6			21.2		
	11	21.5			22.6		
	12	22.1			23.1		

Notation: SD—standard deviation.

**Table 4:** Results of the measurements performed for Ti samples.

Group	Sample	Surface Area (mm^2^)	Area of the Affected Surface, as Obtained with OCT (mm^2^)		Area of the Affected Surface, as Obtained with SEM (mm^2^)				Total Area Affected, as Obtained with OCT (mm^2^)	Total Area Affected, as Obtained with SEM (mm^2^)
CV	2	55.38	3.06	5.8	1.84	1.12	5.22	1.74	8.86	9.96
	4		4.2	4.37	4.45	2.44	1.05	1.46	8.57	9.40
Er:YAG SSP mode	9		6.45	1.76	2.2	1.18	2.76	2.1	8.21	8.24
	10		4.2	2.64	3.84	1.52	0.5	1.61	6.84	7.47
	11		2.15	2.7	1	0.8	0.65	0.92	4.85	3.37
	12		4.01	9.83	4.9	3.31	3.26	4.44	13.85	15.91
Er:YAG QSP mode	17		4.86	3.9	1.55	1.64	2.02	1.8	8.76	7.01
PDT and Er:YAG	13		7.48	9.03	3.48	4.07	4.71	3.24	16.51	15.5
	14		8.38	6.14	3.63	4.18	3.4	3.19	14.52	14.4
	15		5.08	2.22	1.79	2.54	1.98	1.3	7.30	7.61
	16		2.04	2.42	1.04	0.7	1.26	1.75	4.46	4.75

**Table 5:** Results of the measurements performed for the Zr samples.

Group	Sample	Surface Area (mm^2^)	Area of the Affected Surface, as Obtained with OCT (mm^2^)		Area of the Affected Surface, as Obtained with SEM (mm^2^)				Total Area Affected, as Obtained with OCT (mm^2^)	Total Area Affected, as Obtained with SEM (mm^2^)
CV	2	55.38	3.87	1.79	0.24	1.19	2.31	1.74	5.66	5.48
	4		2.61	1.42	0.67	0.81	1.02	0.53	4.03	3.03
Er:YAG SSP mode	9		2.65	3.75	1.54	1.83	0.97	1.28	6.40	5.62
	10		2.13	4.34	1.71	2.14	1.87	0.43	6.47	6.15
	11		1.17	2.93	0.55	1.23	0.84	0.73	4.20	3.35
	12		5.64	5.01	2.05	2.54	3.65	1.29	10.65	9.53
Er:YAG QSP mode	17		4.2	0.69	0.64	1.21	1.03	1.37	4.89	4.25
PDT and Er:YAG	13		5.64	4.11	2.23	1.47	0.88	3.84	9.74	8.42
	14		1.36	2.91	0.95	1.4	2.01	1.19	4.27	5.55
	15		3.57	4.06	1.61	2.37	2.31	1.12	7.63	7.41
	16		5.35	4.36	1.9	4.19	2.56	3.02	9.71	11.67

**Table 6:** Comparison of the OCT and SEM measurements.

	Characteristics of the Affected Surface, as Obtained with OCT (mm^2^)	Characteristics of the Affected Surface, as Obtained with SEM (mm^2^)
Mean (mm^2^)	4.0064	3.9889
Mean Absolute Deviation (mm^2^)	1.5636	1.7441
Standard Deviation (SD) (mm^2^)	2.049	2.1348
Standard Error (SE) of the Mean (mm^2^)	0.3089	0.3218

**Table 7:** Comparison between the conventional method (CV) and Er:YAG or PDT and Er:YAG from the point of view of the impact on the sample’s surface.

		CV (x_1_)	Er:YAG (x_2_)	PDT and Er:YAG (x_3_)	
Sample measurements		3.06	6.45	7.48	
		5.8	4.2	8.38	
		4.2	2.15	5.08	
		4.37	4.01	2.04	
		3.87	1.76	9.03	
		1.79	2.64	6.14	
		2.61	2.7	2.22	
		1.42	9.83	2.42	
		2.96	3.38	7.55	
		6.96	2.11	7.95	
		8.89	1.08	7.81	
		2.51	1.57	6.59	
		1.43	8.21	4.33	
		4.05	7.7	3.28	
		1.48	5.36	1.74	
		1.55	4.86	3.01	
Mean (mm^2^)		3.55	4.25	5.31	
Mean Absolute Deviation (mm^2^)		1.653	2.11	2.3	
SD (mm^2^)		2.086	2.542	2.503	
Pair	Z	SE	Critical value	*p*-value	*p*-value/2
x_1_-x_2_	0.7829	4.9496	11.8489	0.4337	0.2168
x_1_-x_3_	1.9635	4.9496	11.8489	0.04958	0.02479
x_2_-x_3_	1.1806	4.9496	11.8489	0.2377	0.1189

**Table 8:** Comparison between Ti and Zr samples.

	Ti Samples	Zr Samples
Mean (mm^2^)	9.3795	6.5505
Mean Absolute Deviation (mm^2^)	3.1831	2.0324
SD (mm^2^)	3.8865	2.4234
SE of the Mean (mm^2^)	0.8286	0.5167

## Data Availability

Data supporting reported results can be obtained from the corresponding authors.
